# Trajectories of socio-ecological systems: A case study in the tropical Andes

**DOI:** 10.1007/s13280-024-02002-x

**Published:** 2024-04-10

**Authors:** Linda Berrio-Giraldo, Clara Villegas-Palacio, Santiago Arango-Aramburo, Lina Berrouet

**Affiliations:** 1 Departamento de Geociencias y Medio Ambiente, Facultad de Minas, Universidad Nacional de Colombia–Sede Medellín, Cra 80 No 65-223, Bloque M2-301, Medellín, Colombia; 2 Departamento de Ciencias de la Decisión y la Computación, Facultad de Minas, Universidad Nacional de Colombia–Sede Medellín, Cra 80 No 65-223, Bloque M8, Medellín, Colombia; 3 Grupo de Investigación en Ecología Aplicada, Escuela Ambiental, Facultad de Ingeniería, Universidad de Antioquía, Calle 67 # 53 – 108, Medellín, Antioquia Colombia

**Keywords:** Ecosystem services, Policy simulation, Scenario simulation, System dynamics, Socio-ecological system

## Abstract

Scenario and policy assessments in socioeconomic and environmental studies face significant challenges in socio-ecological systems (SES). There are a limited number of studies that have looked at the impact of different scenarios within integrated approaches, and many have used a static approach with a single driver of change. The present work analyzes the SES dynamics for a strategic basin in the Colombian Andes when implementing and analyzing scenarios and policies related to land cover and land use change using a system dynamics simulation model. The model includes natural, ecosystem services, sociocultural, and economic components. Scenarios and policy options are analyzed both individually and jointly to identify synergies or trade-off effects between the different SES components. The results showed the different trajectories of the socio-ecological system according to the cases studied, and its impact on different variables in the analyzed components. Some counterintuitive effects were also identified, such as the importance of intrinsic motivations in decision-making processes, and determinants in land management and policy design.

**Supplementary Information:**

The online version contains supplementary material available at 10.1007/s13280-024-02002-x.

## Introduction

The current pattern of consumption and use of ecosystem services (ES) is affecting the properties and attributes of ecosystems that determine their ecological functioning. Hence, nature’s ability to contribute to people’s well-being continues to significantly decline, implying danger to the economy, livelihoods, food security, and quality of life since it is not possible to guarantee the conditions for the development of societies (IPBES 2019). To safeguard the long-term sustainability of ecosystems, natural capital needs to be adequately managed (Galychyn et al. 2022). The above should recognize the interdependence and the feedback between society and nature and the dynamics of the processes immersed in a territory as a basis for sustainable land planning in the medium and long terms (Vázquez-González et al. 2021; Rodriguez et al. 2021). The socio-ecological system (SES) approach emerged as a framework for understanding the interactions between the social and natural systems of a territory (Collins et al. 2011). Its use provides valuable information for designing strategies to prevent, control, and minimize the negative impacts on life support systems that may result from human activities and the unwanted trajectories of an SES (Nel et al. 2022).

Previous studies have attempted to operationalize the SES framework to analyze different environmental processes or problems (Gomez-Santiz et al. 2021). However, the inclusion of interdependency and feedback mechanisms between natural and social systems and the explicit recognition of delays are still not considered in the implementation of operationalization of decision-making processes of SES management (Drechsler 2020). There is a lack of studies that, through the operationalization of the conceptual models of SES, aim to develop tools for supporting policy design and implementation of territorial planning processes (Perevochtchikova 2019).^1^

The SES operationalization is not an easy task. Simulation models have allowed SES analysis to be operationalized and helped identify possible SES trajectories that may be the result of implementing different management strategies or policies and climate change scenarios (Gotts et al. 2018). Previous analyses of the impact of different scenarios with integrated approaches are scarce in the literature and most of them focused on understanding the effects of the drivers of change on the natural system (Drechsler 2020; Mengist et al. 2020; Ruiz Agudelo et al. 2020; Villamayor-Tomas et al. 2020). A recent literature review found that 74% of the studies that quantified and analyzed ES and SES used a static approach (Obiang Ndong et al. 2020). A dynamic analysis is important because of the different temporal and spatial scales in which processes occur (Dawson et al. 2010; Redondo et al. 2019). Complex systems usually have hidden consequences that are only revealed through a quantitative dynamic simulation. To date, most studies include one driver of change (Pham et al. 2019; Morán-Ordóñez et al. 2020). However, drivers of change are not isolated from each other; hence, they can achieve effects that could be synergistic, complementary, or a trade-off in a territory. Therefore, including more than one driver of change in the analysis is a current challenge in analyzing the SES trajectories.

This manuscript describes SES trajectories using a case study in the tropical Andes under different scenarios and policy options through an integrative system dynamics model previously developed to understand the land cover and land use change (LULCC) processes in a strategic basin (Berrio-Giraldo et al. 2021) in the Colombian Andes.

This study contributes to the literature on SES and LULCC trajectories in two ways. First, we evaluate scenarios and policy options with an integrated approach that includes both the natural and social systems and the interactions generated between them. Second, this study contributes to the design and evaluation of policies and strategies in the planning processes of a territory by demonstrating the impacts over time of scenarios and policies on different variables such as land covers, ES of interest, and other types of variables of a social and economic character.

The remainder of this paper is structured as follows: Section two (2) briefly presents the case study, the model used, and details of the scenarios and policy options considered in this work; Section three (3) discusses the simulation results; and Section four (4) provides the policy implications and conclusions.

## Materials and methods

### Study area

**Fig. 1:**
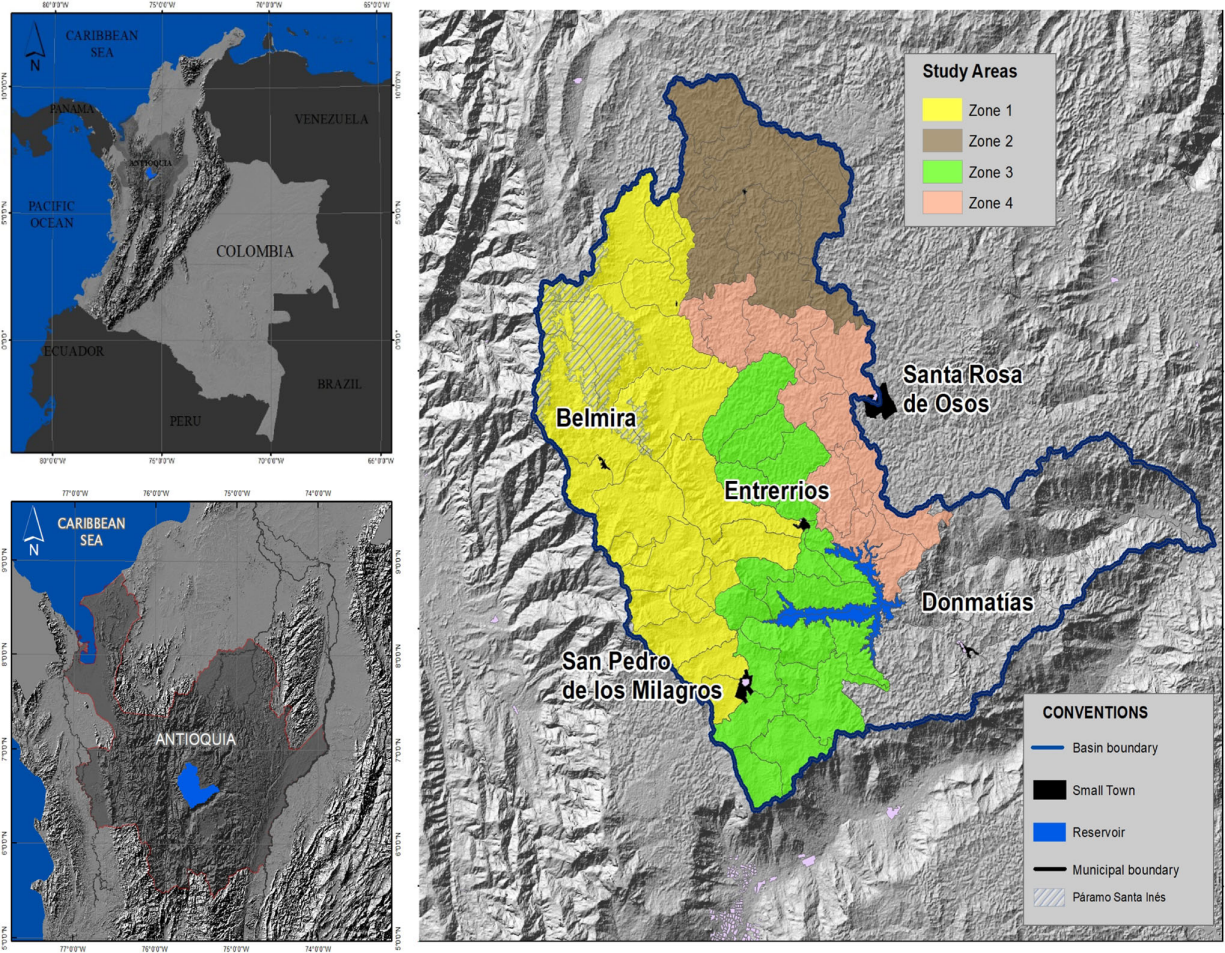
Location and analysis areas for the RGB, Colombia. Adapted from Machado et al. (2019)

The Rio Grande Basin (RGB) is located in the northwest region of Colombia (Fig. 1) with an area of 127,986.3 ha. The RGB is strategic given that it is a source of dairy products for different areas of the country; it has strategic ecosystems, such as paramo and oak forests, and the Riogrande II reservoir is located within the basin, which holds the main water supply of the metropolitan area of Aburrá Valley, the second largest populated center in Colombia,^2^ and electricity generation. At the same time, the basin has been classified as vulnerable to the effects of climate change (García Múnera et al. 2016).

In the basin, there are different government plans, programs, and regulatory instruments for environmental conservation (CORANTIOQUIA and UNAL 2015). For the modeling process, we selected the territory upstream of the reservoir and divided it into four zones (Fig. 1). In Zones 1 and 2, the conservation processes result from formal regulations and community initiatives, such as *Distrito de Manejo Integrado* (DMI),^3^ and *Sistema Local de Áreas Protegidas* (SILAP),^4^ respectively. The community is actively involved in conservation issues in both areas. By contrast, in Zones 3 and 4, conservation processes are scarce, and communities are not active in conservation issues (Villegas-Palacio et al. 2020).

### The model

We selected the system dynamics model recently developed by Berrio-Giraldo et al. (2021) and later refined by Builes et al. (2022) for the SES trajectory analysis. The model was built to understand the LULCC processes for the RGB SES. It has four components: natural, economic, ES, and sociocultural modules within a regulatory and policy framework. The model proposed by Berrio-Giraldo et al. (2021) had as its objective the analysis of the ES of hydrological regulation and erosion control, where it only focused on the quantification of the supply of surface water in the first mentioned service. However, the processes of LULCC are also determinant in the quality of the water resource and limiting its use. Therefore, the ES component was strengthened in the model with the inclusion of the water quality characteristic in the modeling for this study. This section briefly describes the modules, the integration between these modules, and the mentioned novelty.

The natural component includes the three-level (also called stock) variables of *Croplands* (C), *Pasturelands* (P), and *Forests* (F) and models the number of hectares of each land cover according to the conversion rates. The ES component uses an open hydrological simulation model to estimate the S*urface Water Supply* (Velez 2001). This component also employs the Universal Soil Loss Equation to provide information on *Soil Loss* as a proxy for *Erosion Control* (Wishmeier and Smith 1978). As a contribution to the model developed by Berrio-Giraldo et al. (2021), we included the ES of the *Water Quality* through the *Index of Pollution by Suspended Solids* (IPSS). The constant expansion of the agricultural and livestock frontier has reconfigured the landscape (Bourgoin et al. 2020), generating a decrease in the water flow regulation processes and an increase in runoff and erosion rates causing disturbances in the water quality due to loss of soil and nutrients (Suescún et al. 2017; Alamdari et al. 2022). The contribution of sediments in water streams and the decrease in reservoir capacity to sediment accumulation are environmental problems associated with the basin.

In the model, soil loss processes determine the *concentration of suspended solids* in the water streams and the IPSS. The IPSS is a *reduction factor* (RF) for the availability of water for human consumption: high IPSS values limit access to its use. Tamayo (2022) stated that the *concentration of suspended solids* (${C}_{s}$) is given by Eq. (1), which is an approximation by Clarke (1996) for the concentration of suspended sediments using the slurry mass flow and sedimentation equations:

1 $${C}_{s}={\rho }_{s} \left(1-n\right){k}_{E}{\left({\tau }_{0}-{\tau }^{*}\right)}^{N}\left(\frac{18\mu }{\left({\rho }_{s}- \rho \right)g{D}_{P}^{2}}\right),$$

where $\mu$ refers to the water viscosity; ${\rho }_{s}$ and $\rho$ are the densities of sediment and water, respectively; $g$ is gravity; and ${D}_{P}$ is the average particle diameter. Although Clarke (1996) estimated erosion using Eq. (2), this can also be obtained through other approximations, such as the Universal Soil Loss Equation used in the model (Tamayo 2022). The sediment supply received by the water channel and the ability to transport from it can be studied from the movement type, flow measurement method, and sediment source (Osorio Yepes 2016).

2 $$E={\rho }_{s} \left(1-n\right){k}_{E}{\left({\tau }_{0}-{\tau }^{*}\right)}^{N}$$

With the ${C}_{s}$ information, the IPSS is estimated through the proposal made by Ramirez et al. (1997) [Eq. (3)]. According to Ramirez et al. (1997), the IPSS varies between 0 and 1. The index is considered to take values of 1 in cases where the concentration exceeds 340 mg/l. No contamination by suspended solids is considered when the concentration is less than 10 mg/l. The IPSS has an associated pollution category; hence, the RF shall be equal to the value obtained in the IPSS, and the *water available for use* will finally be estimated using Eq. (4).

3 $$ IPSS = \left\{ {\begin{array}{*{20}l} {If\,C_{s} < 10 \,{\raise0.7ex\hbox{${mg}$} \!\mathord{\left/ {\vphantom {{mg} l}}\right.\kern-0pt} \!\lower0.7ex\hbox{$l$}};the\,IPSS = 0} \\ { - 0.02 + 0.003*C_{s} \,\left( {{\raise0.7ex\hbox{${mg}$} \!\mathord{\left/ {\vphantom {{mg} l}}\right.\kern-0pt} \!\lower0.7ex\hbox{$l$}}} \right)} \\ {If\, C_{s} > 340\, {\raise0.7ex\hbox{${mg}$} \!\mathord{\left/ {\vphantom {{mg} l}}\right.\kern-0pt} \!\lower0.7ex\hbox{$l$}};\,then\, IPSS = 1} \\ \end{array} } \right. $$

4 $$Water\,available\,for\,use= Surface\,Water\,Supply *(1-{\text{RF}})$$

The economic component includes the three stock (level) variables of *Productivity of tamarillo* (Prod Tam), *Productivity of potato* (Prod Pot), and *Number of cattle per hectare* (Cat per Ha).^5^ The three stocks are affected by the soil loss calculated in the ES component and a percentage of reduction $\%SoilLoss.$ The *Net Economic Benefits* of the productive activities associated with each land cover are calculated as the difference between the *Total Income* and the *Total Costs*. The *Total Costs* include the operating and investment costs. The *Total Income* is obtained with the information of *Productivity* and hectares of each land cover.

The sociocultural component includes social, cultural, and political elements that allow the capture of interactions between social and natural systems and influence land use decision-making processes. Previous works in the study area have found that small producers are dedicated to livestock activity because of the following reasons: (1) they culturally identify themselves with this activity; (2) it was the economic activity of their ancestors; or (3) they feel safe given that there are companies or cooperatives within the basin that will buy their produce (Vargas 2020). Past studies have also found that existing forests in highlands are conserved by the link with water springs and, in other cases, by the perception of the presence of the environmental authority that regulates and monitors exploitation activities (CORANTIOQUIA and UNAL 2015). Therefore, the variables of *Perception of Security* in product commercialization associated with land use, *Tradition* of each economic activity, *Population growth*, and *Water demand* for each sector were considered in this component. The component also included the second-level variables of the SES framework proposed by McGinnis and Ostrom (2014), which are *trust*, *leadership*, surveillance and sanction rules, governmental organizations, and *community organizations* (Builes et al. 2022). In addition, three types of environmental policy instruments, namely command and control, economic, and informative, were taken and connected with the key elements of social capital (Builes et al. 2022). These variables were included in the RGB SES system dynamics simulation model and are determinants in the land cover selection decision-making process, specifically in the traditional decision criterion that is part of the attractiveness variable (for more details about this component see Builes et al. (2022)).

This model integrates different modules using some connecting variables. One of these variables is *Attractiveness*, which helps us identify the degree to which landholders are attracted to each land cover. *Attractiveness* (${\propto }_{i})$ is obtained from a multi-dimensional discrete choice model proposed by McFadden (2001), which compared the criteria of *Net Economic Benefits*, *Perception of Security*, and *Tradition* (output variables of the economic and sociocultural modules) of each land cover. *Attractiveness* determines the conversion rates between land cover and what is used in the natural module.

The model had 13 stock (level), 146 auxiliary variables, and 141 parameters.^6^ The model parameters were obtained from previous research in the region (Berrouet et al. 2019; Builes et al. 2022; CORANTIOQUIA and Universidad Nacional de Colombia 2012; CORANTIOQUIA and UNAL 2015; García-Leoz et al. 2018; Machado et al. 2019; Ramirez et al. 2018; Suescún et al. 2017; Universidad de Antioquia and CORANTIOQUIA 2009; Villegas-Palacio et al. 2020), government and local database (i.e., Antioquia Statistical Yearbook), literature review associated with each of the processes that have been included in the modeling, and finally, semi-structured interviews with experts from the components and knowledgeable about the basin to capture missing information. The model was validated for both structure and behavior and calibrated with the Powersim Studio Version 10 Academic optimization tool (Powersim Software AS) between 1986 and 2015. The structure validation considered the model scope, extreme conditions, and dimensional consistency (Sterman 2000), while the behavior validation considered a comparison with historical data for some time. Likewise, the validation by academics who performed studies in this basin is considered to corroborate the behavior of the SES variable and sensitivity analysis through semi-structured interviews and guided workshops of each of the components (ES, natural, sociocultural, and economic components. This validation of structure and behavior followed the same steps carried out for the base model and the same historical information was compared, therefore, for greater detail you can review the research developed by Berrio-Giraldo et al. (2021).

### Simulation of scenarios and policy options

This section describes the simulation of different scenarios and policy options^7^ analyzed in this study from 2016 until 2040. This time window was selected considering that, in some cases, the effects of the scenarios and policies will be observed only after some time due to the SES delays.^8^

#### RGB Scenarios

The following scenarios were considered for our analysis: (i) Business As Usual (BAU); (ii) exclusion of intrinsic motivations for conservation; and (iii) climate change. Table 1 summarizes the different scenarios and corresponding parameter values.

**Table 1:** Summary of the analyzed SES scenarios

Scenario	For 2040	Parameters
BAU	BAU	IC: 50% Conservation income: 0 USD
Intrinsic motivations	Forest tradition in conservation processes	Forest tradition: 0
Climate change	Temperature and precipitation increase (Case 1)	Increase in temperature of 0.04 °C/year and precipitation of 7.1 mm/year
	Temperature increase and precipitation decrease (Case 2)	Increase in temperature by 0.04 °C/year and decrease in precipitation of 7.4 mm/year

BAU assumes that the pattern of precipitation, average temperature, prices, and costs of products do not change over time. For our simulation, institutional capacity (IC) is the ability of institutions to formulate, coordinate, implement, and monitor command and control policies. IC is related to the effectiveness of environmental authorities (Colino et al. 2007). In BAU, there is a 50% effectiveness of the environmental authority in the implementation and enforcement of environmental policies (UNAL et al. 2015). Also, we do not consider conservation income generated by strategies such as payments for environmental services in this scenario.

Land use decisions in the basin are determined by economic and social variables (i.e., social norms and cultural and political factors) (Tsai et al. 2015). Previous studies in the basin have found that existing forests in high areas are conserved by their link with water sources and, in other cases, by the perception of the presence of an environmental authority that regulates and monitors exploitation activities (CORANTIOQUIA and UNAL 2015). We evaluated the implications of the absence of intrinsic motivations in conservation in the modeling, which is considered as the *Intrinsic Motivations* (refers to all the cultural and internal factors that influence the landowner's decision-making process) variable in the model, to compare the SES trajectories and determine its importance in the planning processes and effectiveness of strategies for the RGB.

We considered the effects of climate change in the last scenario. Climate change is another driver of change for the basin (García Múnera et al. 2016). Recent studies documented increasing trends in a series of average and minimum temperatures that have been estimated at 0.4 °C/decade (Pérez et al. 1998; Carmona et al. 2014). A combination of increasing and decreasing trends was observed in precipitation throughout the country, and these variations were estimated at 7.4 mm/yr (Carmona et al. 2014). Our proposed climate change scenarios were based on these findings. The first case is related to temperature and precipitation increase. The second case is associated with temperature increase and precipitation decrease (Table 1).

#### Policy Options of RGB

In the policy option category, the possible basin trajectories were considered in terms of the different management strategies that can be implemented in the basin or national-level policies that directly affect the SES. Table 2 presents the analyzed category and its parameters, which are (i) Change in Institutional Capacity, (iii) Free Trade Agreements (FTA), and (iii) Payment of Ecosystem Services.

**Table 2:** Summary of the considered SES policy options

Policy options	For 2040	Parameters
Institutional capacity	High Institutional Capacity	IC: 90%
	Low Institutional Capacity	IC:10%
Free trade agreements	FTA	Decrease by 35% in milk price
Payment of ecosystem services	Payment of Ecosystem Services (PES)	Conservation income: 114 USD

We considered cases where the effectiveness of the environmental authority increased and decreased. Accordingly, we evaluated the scenario of a strengthening (10%) and a weakening (90%) IC as a proxy for the stated scenarios.

We also considered FTA, which are multilateral foreign policy instruments that countries use to consolidate, expand access to their products, and eliminate tariff and non-tariff barriers. This instrument allows the establishment of cooperation mechanisms between contracting parties. Colombia has signed 14 treaties in recent years; however, the last couple of years have witnessed a debate in the dairy sector given the possibility of a new agreement with New Zealand and Australia.^9^ For this possibility, a 35% price decrease is assumed, which is the estimation of the Colombian Association of Milk Processors^10^ in case the agreement is signed.

The Payment of Ecosystem Services (PES) schemes are defined as voluntary transactions between users and service providers conditioned to the agreed natural resource management rules to generate off-site services (Wunder 2015). In the RGB, the environmental authority estimated the incentive to pay for ES through the opportunity cost method (CORANTIOQUIA and UNAL 2015). The average value to pay was 114 USD/ha year for 2015. This information was included in the model as an income associated with the forest cover for conservation.

## Results and discussion

This section presents the simulated trajectories for assessing the impact of the selected scenarios and policy options on the SES. We will compare each case with BAU and discuss the policy implications and its importance for this research area.

### Scenario impact on the SES trajectories

**Fig. 2:**
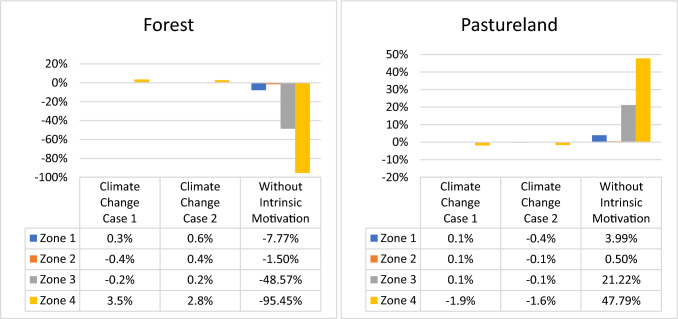
Percentage change in forest and pastureland cover in 2040 for scenarios compared to Business As Usual (BAU) scenario. The climate change cases 1 and 2, and without intrinsic motivation scenarios were considered in this figure

The effects in the land cover trajectories for each zone within the basin varied according to the evaluated scenario. We evaluated the impact of removing the intrinsic motivation variable in the SES modeling to observe its effect on the SES trajectory, which, to our knowledge, had not yet been considered in the literature. Intrinsic motivation is a key component in the RGB that is viewed as a criterion in the land-use decision-making process of landholders. The results indicated a deforestation increase (Fig. 2). The differences observed between the zones were related to the characteristics of the community organization and their conservation processes.

The variations in Zones 1 and 2 were low because the community initiatives for the protection and conservation of the forest cover of these zones are included in formal processes, such as DMI and part of SILAP (España 2020). The extent of forests was controlled by the command and control policies monitored by environmental institutions, and no major changes were expected in the absence of the intrinsic motivation variable. In contrast, Zones 3 and 4 showed higher variations because part of the forest cover areas in these zones is a result of the isolated and unorganized initiatives (not included in formal conservation processes) associated with the importance of water sources, value of existence, and motivations of inheritance (CORANTIOQUIA and UNAL 2015).

This result validates the importance of taking sociocultural elements into account in designing and implementing policies within an SES, since they are crucial in the sustainable management of the territory, as stated by Alexander et al. (2016), Folke et al. (2005) and Ostrom (2009). Ignoring the social dynamics in the implementation can negatively affect SES on time because it cannot guarantee the potential supply of ES and well-being for communities. After all, this affects the effective design of sustainable territory management strategies (Rode et al. 2015; Gunderson et al. 2016). The variables erosion, concentration of suspended solids, and the reduction factor (which translates into quality) are also susceptible to changes since this scenario impacts the land cover trajectories (Table 3). The changes in erosion are proportional to the levels of deforestation according to the relationship that exists with the land cover (Suescún et al. 2017; García-Leoz et al. 2018). The higher the erosion levels, the lower the water quality, considering that it is reduced by the presence of suspended solids. For Zones 1 and 4, the reduction factor is higher (19.4%) compared to Zones 2 and 3 (15.6%) because the concentration of suspended solids is higher for zone 4.

**Table 3:** Impacts of scenarios and policy options evaluated on some variables of SES

	Without intrinsic motivation (%)				FTA (%)				Low institutional capacity (%)				High institutional capacity (%)				PES (%)			
	Zone 1	Zone 4	Zone 2	Zone 3	Zone 1	Zone 4	Zone 2	Zone 3	Zone 1	Zone 4	Zone 2	Zone 3	Zone 1	Zone 4	Zone 2	Zone 3	Zone 1	Zone 4	Zone 2	Zone 3
Water erosion	3.6	45.0	0.5	20.2	− 4.5	− 22.6	− 2.9	− 5.2	2.6	1.2	8.6	10.8	− 2.3	− 1.1	− 8.4	− 11.0	− 0.2	− 0.5	− 0.1	− 0.2
Social Perception Risk	N/A	N/A	N/A	N/A	− 76.2	− 43.0	− 88.5	− 93.0	− 57.1	− 43.6	− 3.8	− 4.0	− 91.0	− 40.6	3.8	3.8	− 76.4	− 42.2	0.0	0.0
Intrinsic Motivation	N/A	N/A	N/A	N/A	0.0	− 38.7	− 42.2	− 25.8	0.0	− 49.4	− 10.0	0.0	0.0	− 26.9	0.0	0.0	0.0	− 38.3	0.0	0.0
Net Economic Benefits	4.1	47.7	0.5	21.1	− 147.0	− 136.0	− 172.8	− 169.5	2.6	1.4	9.0	11.1	− 2.3	− 1.2	− 8.8	− 11.3	− 0.2	− 0.6	− 0.1	− 0.2

	Zone 1 and 4	Zone 2 and 3	Zone 1 and 4	Zone 2 and 3	Zone 1 and 4	Zone 2 and 3	Zone 1 and 4	Zone 2 and 3	Zone 1 and 4	Zone 2 and 3
Concentration of SST (mg/l)	15.6	14.0	− 9.7	− 4.5	2.1	10.2	− 2.0	− 10.2	− 0.3	− 0.1
Reduction Factor	19.4	15.6	− 12.1	− 5.0	2.7	11.4	− 2.5	− 11.5	− 0.3	− 0.2

The percentages of variation are obtained from the comparison of the information between 2040 and 2015.

**Fig. 3:**
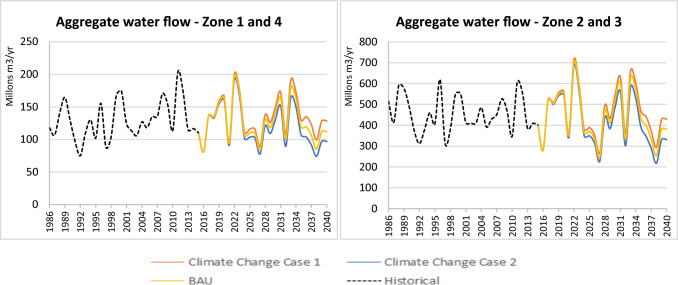
Implications of the climate change scenario on the water available for use

A simulation of the climate change scenarios was performed (Table 2). The obtained results were then compared in terms of the forest cover variations (Fig. 2) and the variable water available for use (Fig. 3). Zones 1 and 4 were more sensitive to the precipitation and temperature variations. The water flow from these areas can increase up to 14.8% in the increased precipitation scenario (Case 1) and decrease up to 13.4% in the decreased precipitation scenario (Case 2). The changes for Zones 2 and 3 (i.e., 12.7 and 12.9%, respectively) were minor compared to those in the other zones for the cases considered in this scenario.

The water flow variations were closely related to the forest hectares in each area. The extent of forest cover in zones 1 and 4 was 33.5 and 32.4%, respectively, of the total area. Zones 2 and 3 covered 38.4 and 47.6% of forest, respectively, due to SILAP implementation. The water flow changes were less in areas with greater forest presence. Zones 2 and 3 presented ecological attributes that favor a better response or adaptation to climatic variability phenomena. The simulations of the climate change scenarios validated the importance of the forest cover level for the ability of a natural system to absorb pressures or pulses and not affect the SES response (IPBES 2019; IPCC 2022). The importance of forests in the result of water available for use was consistent with that found by García-Leoz et al. (2018), who recorded that forests from early stages have a better water regulation capacity for the basin than other land cover vegetations. As the changes in land cover are small, no implications are observed in the other SES variables.

### Evaluation of policy options

**Fig. 4:**
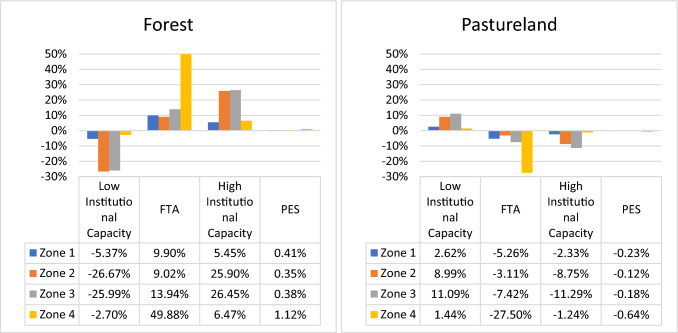
Percentage change in the forest and pastureland covers in 2040 for the policy options compared to BAU. Free Trade Agreements (FTA), Low and High Institutional Capacities, and Payment of Ecosystem Services (PES) were considered in this figure

The policy options considered are the change in the Institutional Capacity (IC), Free Trade Agreements (FTA), and the implementation of the PES. The land cover trajectory depended on whether the effectiveness of the environmental authority increased or decreased. The forest cover area differences can reach up to 26.5% for institutional strengthening and − 26.67% in the case of detrimental capacity when compared with BAU (Fig. 4). The forest cover variations observed for Zones 1 to 3 were the result of formal regulations (e.g., DMI and SILAP) and in Zone 4 of other command and control policies, such as hydric restrictions zones. Zones 2 and 3 showed the highest forest cover variations among the four zones because SILAP had more forest cover in protection and conservation compared to the other command and control figures present in the basin. The DMI and SILAP instruments were created and implemented to protect various ES, mainly water regulation.

The analysis and the measurement of the IC have been a long-term concern, especially for developing countries with high ecosystem richness, such as Colombia (Naidú Duque Cante 2012; Ospina and Chitiva 2016; Ricardo Completa 2016). An institution with capacity deficits in the most critical management processes affects the effectiveness of public policies and their compliance (Ricardo Completa 2016). However, institutional strengthening implies additional costs for implementing and supervising the regulation (Marneffe and Vereeck 2010).

We also observed the effects of the LULCC on other variables, such as erosion control, suspended solid concentration, social capital elements (e.g., social risk perception and intrinsic motivations), and net economic benefits (Table 3). Given the interdependence between systems, this is important when conducting an SES analysis (Gaines et al. 2017). In the case of the IC increase, the pastureland is also reduced which implies lower erosion values, and lower suspended solid concentration in the water streams. As these changes improve the potential supply of the ES analyzed, the social perception of the risk of loss of these services is reduced and, therefore, the motivation to conserve the forests is no longer important for the communities to work on this. Table 3 also shows that the net economic benefits perceived by livestock activity were reduced (i.e., 11.3% for zone 3). These economic losses may affect the purchasing power of holders, the attractiveness of economic activity, or other collateral effects.

In the FTA adoption, all zones showed a forest cover increase (Fig. 2). The variations in the extent of forest cover were 9.9, 9.02, 13.94, and 49.88% for Zones 1 to 4, respectively. The FTA implied a lower milk price compared to BAU and showed a decrease in the net economic benefits of economic activity because the income did not compensate for the production costs^11^ (Table 3). The attractiveness for livestock activity decreased what disincentives the pastureland cover.^12^ Compared to Zones 1 and 2, the changes in Zone 4 were greater than those in the other zones because the zone presented scarce conservation and lower organizational processes concerning the part of communities in conservation issues. With the increase in forest cover under this policy, it is also expected that erosion conditions and concentration of suspended solids will improve and therefore a lower reduction factor (better water quality) as observed in Table 3.

In the PES scenario, the land cover trajectories did not change compared to BAU (Fig. 4) because the payment did not exceed the opportunity cost of the economic activity associated with the pastureland, transaction costs, and intrinsic motivation for ES provision.^13^ Erosion, suspended solid concentration, availability of water for use, and net benefits of agriculture and livestock do not vary because there are no changes in the different land cover (Supplementary Information, Table S1, S2). An improvement is only perceived in the purchasing power of forest landholders who are covered by this type of conservation policy.

The result obtained here regarding the PES implementation was consistent with that found by Velasco Dorado (2019), who concluded that this instrument is not the most suitable for the RGB. The resources that are under plan for investment in the implementation of this voluntary instrument can instead be used to strengthen competent environmental authorities because they affect the forest cover. Note that higher payment values for conservation improve the change rates in the forest cover area. Therefore, considering that the payment value has limits in the implementation process, a cost–benefit analysis must be performed between the PES policy and institutional strengthening concerning the forest cover areas earned.

### Impact of the combination of scenarios and policy options on the SES trajectory

The normal operation of resource management in a territory does not experience isolation scenarios and policies; instead, multiple conditions and policies exist at the same time. Therefore, this study analyzed the combinations of scenarios and policy options to study the impact it would have on the SES trajectory. Four combinations were considered (Table 4) and configured according to the results obtained in Sections “Scenario impact on the SES trajectories” and “Evaluation of policy options” on the changes in land cover and effects in other SES variables.

**Table 4:** Summary of the considered combinations of scenarios and policy options

	Scenario	Policy options	
Combination 1	Climate Change Case 1	High Institutional Capacity	FTA
Combination 2	Climate Change Case 1	Low Institutional Capacity	
Combination 3	Climate Change Case 2	High Institutional Capacity	
Combination 4	Climate Change Case 2	Low Institutional Capacity	

**Fig. 5:**
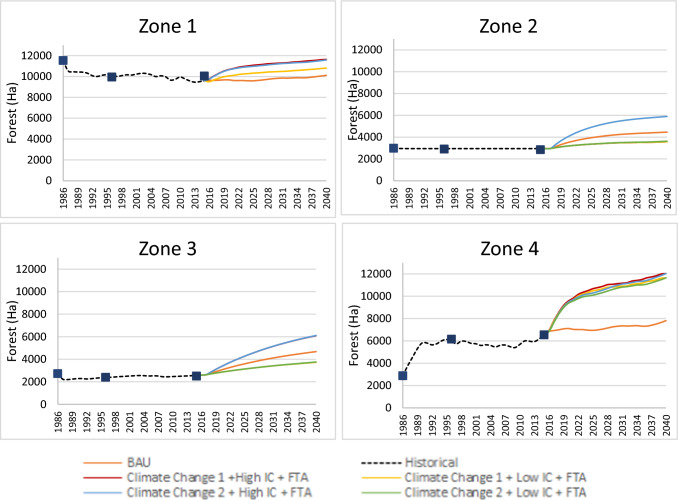
Forest land cover trajectories under the combinations of scenarios and policy options (2015–2040). The square located in the figure represents the points that were considered for the validation of the model concerning land cover

Figure 5 presents the different forest cover trajectories according to the evaluated combinations, in which the impacts were differentiated across zones. In Zones 1 and 4, a forest gain was observed in all combinations compared to BAU. However, we highlight the two following points: (i) the increases in the forest extent were lower in the combinations that considered low IC (combinations 2 and 4); and (ii) Zone 4 showed the strongest change rate when compared to Zone 1, that is, Zone 4 can reach changes of up to 53.98% by 2040 compared to only 15.07% for Zone 1. These results are again led by zone characteristics. The decision-making processes for Zone 4 are mainly determined by the profitability of each of the covers, with grass being the highest, compared to Zone 1. Then, in the results, the price decrease by the FTA prevails despite the low IC considered in the simulated combination. The climate change scenario does not bring changes in forest cover, but it does in the supply of surface water available for use. The increase in forest area implies lower levels of erosion (approximately a 51.9% reduction for zone 4 in the simulated combination 4), lower concentration of suspended solids (approximately a 43.6% reduction for zone 1 and 4 in the simulated combination 2), and, therefore, less contamination of water courses (approximately a 54.3% decrease in the reduction factor for zone 1 and 4 in the simulated combination 2). The foregoing considers that the grass cover areas are also reduced (Table S1, S2).

In Zones 2 and 3, deforestation processes were observed in two of the simulated combinations, that is, combinations 2 and 4, with similar change rates (i.e., reduction in forest cover of up to 20.29% and 19.75% for Zones 2 and 3, respectively). These deforestation processes demonstrated the relevance of the variables of tradition (associated with livestock activity in this case) in the LULCC processes because, despite the low economic yields (i.e., approximate reductions of 120% in combination 2 and 140% in combination 4), landholders prefer to extend the livestock frontier when the IC of environmental authorities is low (Table S1, S2).

This result validates the importance of considering the different decision-making criteria of landholders in an SES. In the RGB, some motivations were more evident in some zones compared to others. In one case, the extent of the pastureland cover was very sensitive to a decrease in Zones 1 and 4 when the profitability of the livestock activity was affected by the decrease in milk prices, even when the environmental authority did not conduct monitoring and surveillance processes. This result is consistent with the conclusions obtained in the research of (Berrouet et al. 2020; Vargas 2020; Suarez 2020), where they found that for these areas economic interest is prevalent in the decision-making processes of landholders for the definition of land cover and land use. This is mainly because Zones 1 and 2 have a greater representation of large milk producers. On the contrary, the number of hectares of pastureland covered in Zones 2 and 3 was mediated by the elements of heritage and tradition that mark the livestock activity. This coincided with the results found by España (2020), who showed that this type of economic activity remains in time despite attempts to delimit protected areas through SILAP. Similarly, the effects were evident in the other SES variables conceptualized for the RGB model such as water supply available for use, erosion level, suspended solid concentration, and water stream contamination (Table S1, S2).

## Policy implications and conclusions

In this study, by using a system dynamics model, we analyzed the effect of scenarios and policy options in the trajectories of an SES in the Colombian Andes. In contrast to previous literature that focuses on one of the subsystems (Dawson et al. 2010; Mayer et al. 2014; Barton and Gutiérrez-Antinopai 2020), our model included elements of sociocultural, economic, ES, and natural subsystem.

Including these components in an SES model and evaluating different scenarios and policies in isolation together represents the most important methodological novelty of this research work. Understanding the complex system and the possible response trajectories of the SES are essential for designing and implementing sustainable management of the territory. We have shown here how the whole picture of the policy analysis avoids the situation of solving one problem by causing another bigger problem (i.e., when strict conservation strategies without considering their effects on the social system or economic incentive policies without considering their effects on the natural system are applied). Through the implementation of a case study, our results suggest the advantage of the tool with a comprehensive vision for the design of management tools and building resilience for SES.

The RGB is a strategic mountain ecosystem because of the multiple ES it offers, such as the provision of fresh water for drinking and the diverse economic activities in the basin. This represents progress in an area pending work because there are only a few applications developed in the southern hemisphere with different idiosyncrasies, local conditions, and needs (Mengist et al. 2020). Although an improvement in the natural system conditions and ES was analyzed in some of the evaluated scenarios and policy options, it also affected the social system of the SES by decreasing the net economic benefits perceived by the economic activity associated with the pastureland cover. In this work, the changes in different SES variables and information for performing a complete analysis of the decision-making processes can be quantified.

In fact, for the case study, we found both expected and counterintuitive results that are characteristic of complex systems. On the one hand, the result of PES and the behavior of the combined scenarios give us information for the management processes. This is because, for example, instead of allocating or adding instruments without considering the additional effect on system dynamics, we can move scarce resources to unsolicited planning tools, or that may also be ineffective in contexts of institutional weakness, as is the case in our region.

On the other hand, the importance of intrinsic motivations associated with conservation was evident during the modeling process. This result is especially important when evaluating other types of voluntary instruments with economic incentives, for example, the PES as an instrument that has become popular in recent years because of its multiple advantages. In this work, the PES did not observe an increase in forest cover, but it did improve the income received by the landholders who had this land cover. However, the financial sustainability over time of such instruments is low. In Colombia, this type of contract has had a duration ranging from 4 to 10 years. Therefore, landholders could lose this incentive in the long term. This will lead to the risk of eroding the individual ethics of protection and changing the relationship that exists with the environment for the rationality of individual benefit in an SES, consequently generating inefficiency in future.

SES modeling is a field that is in development, so any exercise that can be carried out represents a methodological advance that is useful in the replication process and an advance in the experiences learned to avoid subsequent setbacks. In the same way, this exercise also gives an idea of the information that is necessary to collect from primary and secondary sources to materialize an SES modeling process. The model could be applied in similar geographic contexts where governance processes are similar, and where the services of interest are those considered in this research. Some of the challenges that are important to mention in this area of research include the explicit spatial necessity in conjunction with this dynamic analysis, and the information that is necessary to carry out these modeling processes.

This study includes components of SES, and it is possible to observe changes in these variables over time. However, the spatial variability cannot be captured in the best way despite the classification into zones. Therefore, it is recommended to review the application of this type of model with software compatible with GIS. Similarly, it is noteworthy that the implications for changes in ecosystem services and the other SES variables are only associated with changes in land cover and not with land use practices. This represents a limitation of the model and an opportunity for improvement for further research.

## Electronic supplementary material

Below is the link to the electronic supplementary material.

Supplementary file1 (PDF 777 kb)

## Footnotes

1 The term “operationalization” refers to the process by which a conceptual SES framework is translated into a measurable set of variables, relationships, and indicators linked to each of the systems in the SES (Leslie et al. 2015).

2 According to the projections recorded in 2016 by the Statistical Yearbook of Antioquia, the metropolitan area of Aburrá Valley had 3,866,165 inhabitants in 2017.

3 DMI for its name in Spanish: *Distrito de Manejo Integrado is a land planning tool with a restriction some land uses.*

4 SILAP for its name in Spanish: *Sistema Local de Áreas Protegidas is a biodiversity management instrument.*

5 Information taken from Antioquia Statistical Yearbook for 2016-http://www.antioquiadatos.gov.co/index.php/produccion.

6 For more detail review the supplementary material.

7 Scenarios refer to possible future situations or sets of hypothetical circumstances that could occur, based on different variables or factors that are external to the decision makers. On the other hand, policies are sets of principles, rules or guidelines established to guide decision-making and specific actions in certain areas, with the aim of achieving certain objectives or solving specific problems.

8 Some authors, such as (Sterman 2000), recommend that the simulation should be projected at least 3 times the largest delay that the system has. In this case, this is observed with the process of transition to forest land cover for hydrological regulation purposes, which is 7 years.

9 Information was taken from https://www.portafolio.co/economia/finanzas/analisis-impacto-tlc-estados-unidos-25332. [Retrieved June 10, 2019].

10 Information was taken https://www.semana.com/pais/articulo/asoleche-se-opone-a-los-tlc-entre-colombia-y-nueva-zelanda/258943/

11 The decline in the price of milk associated with the FTA is caused by two main reasons: (i) the technical production practices of external dairy markets lower their production costs compared to the traditional systems in which the basin operates; and (ii) elimination of tariff and trade barriers (Rezitis et al. 2021).

12 The benefit reductions can reach differences of up to 172.8% in 2040 (Table 3), thereby affecting the social system of the case study.

13 This investigation simulated different values for the payment and observed changes in the SES trajectory when these are greater than those obtained by the environmental authority. When the payment value is tripled, the changes in the forest cover area begin to become greater than 1.3% for Zones 1 to 3 and greater than 3.7% for Zone 4.
