# Supplementary material for: Trajectories of socio-ecological systems: A case study in the tropical Andes
Source: Ambio. 2024 Apr 10;53(12):1737–51. doi: 10.1007/s13280-024-02002-x (PMC11568092; doi:10.1007/s13280-024-02002-x)
Supplement: Supplementary file 1 — Supplementary file1 (PDF 777 kb) [file 13280_2024_2002_MOESM1_ESM.pdf]

Ambio

Supplementary Information

This supplementary information has not been peer reviewed.

Title: Trajectories of socio-ecological systems: A case study in the tropical Andes

Authors: Linda Berrio-Giraldo, Clara Villegas-Palacio, Santiago Arango-Aramburo, Lina Berrouet

Table S1 describes a large part of the variables that intervened in the simulation model proposed for the Rio Grande basin. It is important to highlight that several auxiliary variables were used as supports in intermediate estimates and others were repeated both for each of the zones or for the type of land cover.

Table S1. Definitions and equations of the variables that were included in the modeling of the SES.

VARIABLE	DEFINITION	EQUATIONS	UNITS	TIPE OF VARIABLE
		**Natural Component**		
Protected Area (AP)	Number of hectares of forests protected per year.	N/A	ha	Parameter
Forest to crop conversion time ($\boldsymbol{TC_{b-c}}$)	Physical time needed to convert forest cover to crop	N/A	yr	Parameter
Crop to forest conversion time ($\boldsymbol{TC_{c-b}}$)	Physical time needed to convert crop cover to forest	N/A	yr	Parameter
Forest to Pasture Conversion Time ($\boldsymbol{TC_{b-p}}$)	Physical time required to convert forest cover to pasture	N/A	yr	Parameter
Conversion time from crop to pasture ($\boldsymbol{TC_{c-p}}$)	Physical time required to convert cropland to pasture	N/A	yr	Parameter
Pasture to forest conversion time ($\boldsymbol{TC_{p-b}}$)	Physical time required to convert pastureland to forest	N/A	yr	Parameter
Conversion time from pasture to crop ($\boldsymbol{TC_{p-c}}$)	Physical time needed to convert pastureland to crop	N/A	yr	Parameter
Decision-making time to convert forest to crops ($\boldsymbol{TD_{b-c}}$)	Time required for the decision-making process to convert forest to cropland	N/A	yr	Parameter
Decision-making time to convert forest to pasture ($\boldsymbol{TD_{b-p}}$)	Time needed for the decision-making process to convert forest to pasture	N/A	yr	Parameter

VARIABLE	DEFINITION	EQUATIONS	UNITS	TIPE OF VARIABLE
Decision-making time to convert crops to forest ($\boldsymbol{TD_{c-b}}$)	Time needed for the decision-making process to convert crop to forest	N/A	yr	Parameter
Decision-making time to convert crop to pasture ($\boldsymbol{TD_{c-p}}$)	Time required for the decision-making process to convert crop to pasture	N/A	yr	Parameter
Decision-making time to convert pastureland to forest ($\boldsymbol{TD_{p-b}}$)	Time required for the decision-making process to convert pasture to forest	N/A	yr	Parameter
Decision-making time to convert pasture to crop ($\boldsymbol{TD_{p-c}}$)	Time required for the decision-making process to convert pasture to cropland	N/A	yr	Parameter
Weight Associated with Real Profit ($\boldsymbol{\beta_{BR}}$)	Weight attributed to the Real Benefits criterion in the decision-making process	N/A	%	Parameter
Weight associated with Tradition/Culture ($\boldsymbol{\beta_{T/C}}$)	Weight attributed to the Tradition/Criteria criterion in the decision-making process	N/A	%	Parameter
Weight associated with Risk ($\boldsymbol{\beta_R}$)	Weight attributed to the Risk criterion in the decision-making process	N/A	%	Parameter
Other area (OA)	Area with land cover other than forest, pasture and cultivation.	N/A	ha	Parameter
Forests/Succesional Vegetation (B)	Quantity of Hectares with land cover of forests and stubble.	$B(t) = B(0) + \int_0^t (TC_{P\to B} + TC_{C\to B} - TC_{B\to P} - TC_{B\to C})\, dt$	ha	Stock (Level)
Croplands (C)	Quantity of Hectares with land cover of croplands.	$C(t) = C(0) + \int_0^t (TC_{B\to C} + TC_{P\to C} - TC_{C\to B} - TC_{C\to P})\, dt$	ha	Stock (Level)
Pastureland (P)	Quantity of Hectares with land cover of pasturelands.	$P(t) = P(0) + \int_0^t (TC_{B\to P} + TC_{C\to P} - TC_{P\to B} - TC_{P\to C})\, dt$	ha	Stock (Level)
Deforestation of Forest to Croplands	Number of hectares of forest that are converted to cropland per year.	$\frac{Max(0 \ll Ha \gg;\ Min(ADC - C; ADD))}{TC_{b-c} + TD_{b-c}}$	ha/yr	Auxiliar

VARIABLE	DEFINITION	EQUATIONS	UNITS	TIPE OF VARIABLE
Deforestation of Forest to Pastureland	Number of hectares of forest that are converted to pastureland per year.	$\frac{Max(0 \ll Ha \gg;\ Min(ADP - P; ADD))}{TC_{b-p} + TD_{b-p}}$	ha/yr	Auxiliar
Susceptible Area to deforest (ADD)	Number of hectares of forest available for deforestation in a period of time.	$B - AP$	ha/yr	Auxiliar
Abandonment of Croplands to Forest	Number of hectares of croplands that are converted into forest by abandonment.	$\frac{Max(0 \ll Ha \gg;\ Min(ADB - B; C))}{TC_{c-b} + TD_{c-b}}$	ha/yr	Auxiliar
Abandonment of Pastureland to Forest	Number of hectares of pasturelands that are converted into forest by abandonment.	$\frac{Max(0 \ll Ha \gg;\ Min(ADB - B; P))}{TC_{p-b} + TD_{p-b}}$	ha/yr	Auxiliar
Change of land cover from Pasture to Crop	Number of hectares of pasture converted to cropland	$\frac{Max(0 \ll Ha \gg;\ Min(ADC - C; P))}{TC_{p-c} + TD_{p-c}}$	ha/yr	Auxiliar
Change of land cover from Crop to Pasture	Number of hectares of crops that are converted to pasture	$\frac{Max(0 \ll Ha \gg;\ Min(ADP - P; C))}{TC_{c-p} + TD_{c-p}}$	ha/yr	Auxiliar
Desired Forest Area (ADB)	Indicative number of hectares of Forest according to the preferences of the decision maker	$\%C_B * AV$	ha	Auxiliar
Desired Crop Area (ADC)	Indicative number of hectares of Crop according to the preferences of the decision maker	$\%C_c * AV$	ha	Auxiliar
Desired area of pastureland (ADP)	Indicative number of hectares of Pasture according to the preferences of the decision maker	$\%C_P * AV$	ha	Auxiliar
Variable Area (AV)	Number of hectares susceptible to change of land cover	$AT - AP$	ha	Auxiliar
Total Area (AT)	Number of total hectares	$B + C + P$	ha	Auxiliar
%Conversion to Forest ($\%\boldsymbol{C_B}$)	Percentage of indicative area of Forest of the total area according to the preferences of the decision maker	$\%C_B = \frac{Exp(\sum_{i=1}^{3} \beta_i P_B)}{\sum_{l=1}^{3} Exp(\sum_{i=1}^{3} \beta_i P_l)}$	%	Auxiliar
%Conversion to Cropland ($\%\boldsymbol{C_c}$)	Percentage of indicative area of Cropland of the total area according to the preferences of the decision maker	$\%C_c = \frac{Exp(\sum_{i=1}^{3} \beta_i P_c)}{\sum_{l=1}^{3} Exp(\sum_{i=1}^{3} \beta_i P_l)}$	%	Auxiliar

VARIABLE	DEFINITION	EQUATIONS	UNITS	TIPE OF VARIABLE
%Conversion to Pastureland (% $\boldsymbol{C_P}$)	Percentage of indicative area of Pasture of the total area according to the preferences of the decision maker	$\%C_P = \frac{Exp(\sum_{i=1}^{3} \beta_i P_P)}{\sum_{l=1}^{3} Exp(\sum_{i=1}^{3} \beta_i P_l)}$	%	Auxiliar
%F	Forest percentage with respect to the total area	$\%F = B/AT$	%	Auxiliar
%P	Percentage of pastureland with respect to the total area	$\%P = P/AT$	%	Auxiliar
%C	Percentage of cultivation with respect to the total area	$\%C = C/AT$	%	Auxiliar
Area total (AT)	Total area of each sub-basin	$AT = B + P + C + OA$	ha	Auxiliar
		**Economic Component**		
%Hectare of Potato (%HP)	Percentage of cultivated area devoted to potatoes	N/A	%	Parameter
% Hectare of Tamarillo (%HT)	Percentage of cultivated area devoted to tamarillo	N/A	%	Parameter
Potato Productivity (PP)	Potato crop yield per hectare	N/A	kg/ha/yr	Parameter
Tamarillo Productivity (PT)	Tamarillo crop yield per hectare	N/A	kg/ha/yr	Parameter
Potato price (Pp)	Potato Price	N/A	$/kg	Parameter
Tamarillo price (Pta)	Tamarillo Price	N/A	$/kg	Parameter
j	The discount rate is a financial factor that is used to determine the value of money over time, either to calculate the present value of future capital or to evaluate investment projects. In the model it is considered as the WACC (interest rate of the cost of capital) for the Colombian case.	N/A	%	Parameter
Life time (TV)	Period that the capital investment lasts	N/A	yr	Parameter
Fixed costs (CF)	They are those that are generated regardless of the number of units of goods or services	N/A	$/ha	Parameter

VARIABLE	DEFINITION	EQUATIONS	UNITS	TIPE OF VARIABLE
	produced, that is, without being linked to the volume of activity.			
Variable costs (CV)	They are those that are incurred only if the activity is carried out and, as a consequence of it, a good or a service unit is generated.	N/A	$/ha	Parameter
Heads of cattle per Hectare (CG)	Cattle head yield per hectare	N/A	vaca/ha	Parameter
Days of lactation per year (DL)	Days in the year that the head of cattle produces milk	N/A	da/yr	Parameter
Milk productivity (PL)	Milk production yield per head of cattle per day	N/A	l/da/vaca	Parameter
Milk price (Pl)	Milk price per liter	N/A	$/l	Parameter
Forest income ($\boldsymbol{I_b}$)	Income received for conservation	N/A	$/ha	Parameter
Real benefits of Agriculture (BRA)	Net Economic Benefits generated by Agriculture associated with cropland cover	$I_{ag} - CT_{ag}$	$/yr	Auxiliar
Livestock Real Benefits (BRG)	Net Economic Benefits generated by Livestock associated with pastureland cover	$I_{ga} - CT_{ga}$	$/yr	Auxiliar
Hectares of potato (HP)	Amount of net hectares devoted to potato	$\%HP * C$	ha	Auxiliar
Hectares of tamarillo (HT)	Amount of net hectares devoted to tamarillo	$\%HT * C$	ha	Auxiliar
Amount of potato (QP)	Amount in kilograms of potatoes per year	$HP * PP$	kg/yr	Auxiliar
Amount of tamarillo (QT)	Amount in kilograms of tamarillo per year	$HT * PT$	kg/yr	Auxiliar
Income per potato ($\boldsymbol{I_p}$)	Income generated by potato production.	$QP * P_p$	$/yr	Auxiliar
Income per tamarillo ($\boldsymbol{I_{ta}}$)	Income generated by tamarillo production.	$QT * P_{ta}$	$/yr	Auxiliar
Total Agriculture Income ($\boldsymbol{I_{ag}}$)	Total income generated by Agriculture.	$I_p + I_{ta}$	$/yr	Auxiliar
Total potato costs per hectare ($\boldsymbol{CT_p}$)	Total costs for potato production per hectare.	CAPEX de papa + OPEX de papa	$/(yr*ha)	Auxiliar
Total costs of tamarillo per hectare ($\boldsymbol{CT_{ta}}$)	Total costs for the production of tamarillo per hectare.	CAPEX de tomate de árbol + OPEX de tomate de árbol	$/(yr*ha)	Auxiliar
Total agriculture costs ($\boldsymbol{CT_{ag}}$)	Total costs associated with Agriculture	$(CT_p * HP) + (CT_{ta} * HT)$	$/yr	Auxiliar

VARIABLE	DEFINITION	EQUATIONS	UNITS	TIPE OF VARIABLE
Capital costs (CAPEX)	Capital investments that generate profit for the producer.	$\frac{j * InvCosts}{1-(1+j)^{-TV}}$	$/ha	Auxiliar
Inversión costs (InvCosts)	It is the monetary expense incurred in the acquisition of the assets necessary to put the project into operation.	N/A	$/ha	Auxiliar
Operating costs (OPEX)	They are the costs necessary to produce a good or service.	$CF + CV$	$/ha	Auxiliar
Bovine Inventory (IB)	Number of total cattle heads	$CG * P$	vaca	Auxiliar
Total amount of milk (QL)	Amount of milk in liters	$IB * DL * PL$	l/da	Auxiliar
Total income from livestock ($\boldsymbol{I_{ga}}$)	Total income generated by Livestock	$QL * P_l$	$/da	Auxiliar
Total Livestock Costs	Total costs associated with livestock	CAPEX de ganadería + OPEX de ganadería	$/da	Auxiliar
Total costs per forest	Represents the cost of operating the forest	0	$/ha	Auxiliar
		**Ecosystem Services Component**		
**Hydrological Component - Quantity**				
Beta ($\boldsymbol{\beta}$)	Constant in the hydrological module	-	-	Parameter
$\boldsymbol{H_{sf}}$	Maximum sheet in mm that can be stored by the leaf surface		mm	Parameter
Moisture at field capacity (HFC)	It refers to the relatively constant amount of water that a saturated soil contains after 48 hours of drainage.	N/A	mm	Parameter
Wilting moisture (HPW)	It is the minimum humidity point at which a plant can no longer extract water from the soil and cannot recover from water loss even if the ambient humidity is saturated.	N/A	mm	Parameter
Coverage water storage capacity (WSCC)		N/A	mm	Parameter

VARIABLE	DEFINITION	EQUATIONS	UNITS	TIPE OF VARIABLE
Potential Evapotranspiration (ETP)	It refers to the amount of water that could be evapotranspired if the availability of water is unlimited.	N/A	mm/yr	Parameter
Saturated hydraulic conductivity (Ks)	It represents the greater or lesser ease with which the soil surface allows water to pass per unit of cross-sectional area in the direction of flow.	N/A	mm/hr	Parameter
Residence time of surface water in soil ($\boldsymbol{Tr_{sup}}$)	Average time a water molecule spends in surface storage	N/A	da	Parameter
Percolation capacity (Kp)	It represents the greater or less ease with which water passes through the pores of the soil.	N/A	mm/da	Parameter
Residence time of sub-surface water in the soil ($\boldsymbol{Tr_{sub-sup}}$)	Average time a water molecule spends in sub-surface storage	N/A	da	Parameter
Underground losses (UL)	It is the amount of water that descends from the "Aquifer" tank through the interior of the soil		mm/yr	Parameter
Residence time of groundwater in the soil ($\boldsymbol{Tr_{subt}}$)	Tiempo medio que una molécula de agua pasa en el almacenamiento subterráneo		da	Parameter
Interception (Int)	Corresponds to a volume of water that does not reach the ground surface, and instead becomes a fraction of the precipitated water that can eventually be evaporated to return to the atmosphere.	$Int(t) = Int(0) + \int_0^t (PT - Ev - WNI)\, dt$	mm	Level
Evaporation (Ev)	Physical process that consists of the slow and gradual transition from a liquid state to a gaseous state of water, after having acquired enough energy to overcome surface tension.	$Ev = Min\left\{ETP * \left(\frac{So}{HU}\right)^{\beta}; So\right\}$	mm/da	Auxiliar
Water not intercept (WNI)	Amount of water that does not manage to remain in the interception tank	$WNI = PT - So$	mm/da	Auxiliar

VARIABLE	DEFINITION	EQUATIONS	UNITS	TIPE OF VARIABLE
Maximum interception capacity (HU)	Maximum capacity of the interception tank and the dual depends on the leaf area index.	$HU = IAF * H_{sf}$	mm	Auxiliar
(So)	Amount of water stored in the first tank	$MIN(PT * \beta; MAX(0; (HU - Int)/TIMESTEP))$	mm/da	Auxiliar
Leaf area index total (IAFt)	A dimensionless measure that is defined as the amount of leaf area per unit area of land surface.	$IAFt = (IAF_{forest} * \%F) + (IAF_{crop} * \%C) + (IAF_{pasture} * \%P)$	-	Auxiliar
Capillary Storage (CS)	It is the tank that represents the interception of rain by the vegetation, the detention of water in depressions in the ground and the water that is retained in the upper level of the soil by capillary forces and that is used by plants for their physiological processes.	$CS(t) = CS(0) + \int_0^t (WNI - ETP - EP)\, dt$	mm	Level
Soil water storage capacity (WSCS)		$HFC - HPW$	mm	Auxiliar
Maximum capillary storage capacity (MCSC)	It is the maximum capillary storage capacity	$WSCS + WSCC$	mm	Auxiliar
Real Evapotranspiration (ETR)	It is the amount of water that is effectively evaporated from the soil surface and transpired by the vegetation cover.	$Min\left(ETP\left(\frac{CS}{MCSC}\right)^{0,6}, CS\right)$	mm/yr	Auxiliar
D1	It is the amount of water that enters the “Capillary Storage” tank	$Min\left(PT\left(1 - \frac{CS}{MCSC}\right)^{2}, MCSC + CS\right)$	mm/yr	Auxiliar
Excess moisture (EP)	It is the amount of water that cannot be intercepted by capillary storage or evapotranspired	PT-D1	mm/yr	Auxiliar
Surface storage (SS)	It is the tank that represents the water that reaches the surface of the land and that drains as surface runoff on the slope.	$SS(t) = SS(0) + \int_0^t (EP - It - ES)\, dt$	mm	Level
Infiltration (It)	It is the amount of water that descends from the "Superficial Storage" tank into the soil	$Min(Ks, EP)$	mm/yr	Auxiliar
$\boldsymbol{Alfa_1}$		$\frac{1}{Tr_{sup}}$	da^{-1}	Auxiliar

VARIABLE	DEFINITION	EQUATIONS	UNITS	TIPE OF VARIABLE
Surface runoff (ES)		$If\left(\left(\frac{SS}{Timestep}+EP-It-Alfa_1*SS\right)\geq 0 \ll \frac{mm}{yr} \gg; Alfa_1*SS; EP+\frac{SS}{Timestep}-It\right)$	mm/da	Auxiliar
Subsurface storage (FW)	It is the tank that represents the water that accumulates as groundwater while it circulates in the direction of the slope through the interior of the upper layer of the soil.	$FW(t)=FW(0)+\int_0^t (It-Pt-ESS)\,dt$	mm	Level
Percolation (Pt)	It is the amount of water that descends from the "Sub-surface storage" tank through the interior of the ground	$Min(Kp, Infiltration)$	mm/hr	Auxiliar
Sub-surface runoff (ESS)		$If\left(\left(\frac{FW}{Timestep}+It-Pt-Alfa_2*FW\right)\geq 0 \ll \frac{mm}{yr} \gg; Alfa_2*FW; It+\frac{FW}{Timestep}-Pt\right)$	mm/da	Auxiliar
$\boldsymbol{Alfa_2}$		$\frac{1}{Tr_{sub-sup}}$	da^{-1}	Auxiliar
Aquifer (Aq)	It is the tank that represents the storage of water in the aquifer and that drains as base flow towards the drainage network.	$Aq(t)=Aq(0)+\int_0^t (Pt-UL-FB)\,dt$	mm	Level
$\boldsymbol{Alfa_3}$	Average time a water molecule spends in underground storage	$\frac{1}{Tr_{subt}}$	da^{-1}	Auxiliar

VARIABLE	DEFINITION	EQUATIONS	UNITS	TIPE OF VARIABLE
Base flow (FB)		$If\left(\left(\frac{Aquifer}{Timestep}+Pt-Alfa_3*Aquifer-UL\right)\geq 0 \ll \frac{mm}{yr}\gg;\ Alfa_3*Aquifer;Pt+\frac{Aquifer}{Timestep}-UL\right)$	mm/da	Auxiliar
Surface wáter supply		$ES+ESS+FB$	m^3/seg	Auxiliar
**Water Erosion Component**				
Erodability Factor (K)	This factor represents the susceptibility that a soil exhibits to erosion as a product of some of its intrinsic characteristics.	N/A	N/A	Parameter
Topographic Factor (LS)	This factor is calculated as the product of two relief characteristics already analyzed from the perspective of soil losses: the length of the slope (L) and its inclination or slope (S).	N/A	N/A	Parameter
Vegetation factor (C)	This factor includes the influence that vegetation has on soil losses, controlling both the kinetic energy of raindrops as they fall on the ground surface, and the speed of runoff water, an effect that had already been treated as a regulation.	N/A	N/A	Parameter
Driving Practices Factor (P)	This factor includes the effect that different soil conservation practices have on soil losses.	N/A	N/A	Parameter
Erosivity Factor (R)	This factor is defined as the product of two rain characteristics that explain the impact it has on the generation of the erosion process. The characteristics are kinetic energy and maximum intensity.	$If\ (PT>1240 \ll \frac{mm}{yr}\gg;\ -8{,}831+\ 0{,}0071263*PT;\ 0{,}00001193*PT\text{^}1{,}70148)$	N/A	Auxiliar

VARIABLE	DEFINITION	EQUATIONS	UNITS	TIPE OF VARIABLE
Potential Water Erosion (P Erosion)	It is the erosion generated by the combined effect of several physical factors such as the climate, the topography of the land and the material that makes up the soil of the place.	$R * K * LS$	ton/(ha*yr)	Auxiliar
Current Water Erosion	It is the erosion generated after incorporating the use of the land.	$P\ Erosion * C * P$	ton/(ha*yr)	Auxiliar
		**Socio-Cultural Component**		
Tradition associated with agriculture	Tradition level of the area associated with agriculture. This variable is related to the values of non-use of the ecosystem services provided by the crop land cover.	N/A	N/A	Parameter
Tradition associated with livestock	Tradition level of the area associated with pasture. This variable is related to the values of non-use of the ecosystem services provided by the pastureland cover.	N/A	N/A	Parameter
Incentives for conservation	Existence of incentives that are in force in the area for conservation	N/A	N/A	Parameter
Incentives for agriculture	Existence of incentives that are in force in the area for agriculture	N/A	N/A	Parameter
Livestock Incentives	Existence of incentives that are in force in the area for livestock	N/A	N/A	Parameter
Presence of economic institutions for conservation	Existence of economic institutions that promote conservation activities	N/A	N/A	Parameter
Presence of economic institutions for agriculture	Existence of economic institutions that promote the marketing of agricultural products	N/A	N/A	Parameter
Presence of economic institutions for livestock	Inventories of economic institutions that promote the commercialization of dairy products	N/A	N/A	Parameter

VARIABLE	DEFINITION	EQUATIONS	UNITS	TIPE OF VARIABLE
Population growth rate		N/A	%	Parameter
Water demand for electricity generation with return	Amount of water required in the basin for electricity generation with return	N/A	m^3/yr	Parameter
Water demand for electricity generation without return	Amount of water required in the basin for non-return electricity generation	N/A	m^3/yr	Parameter
Water demand Industrial Sector	Amount of water required in the basin for the industrial sector	N/A	m^3/yr	Parameter
Water demand Services Sector	Amount of water required in the basin for the service sector	N/A	m^3/yr	Parameter
Water demand for Pig Farming	Amount of water required in the basin for the pig sector	N/A	m^3/yr	Parameter
Poultry Water Demand	Amount of water required in the basin for the poultry sector	N/A	m^3/yr	Parameter
Aquaculture water demand	Amount of water required in the basin for the aquaculture sector	N/A	m^3/yr	Parameter
Average Water Requirement for Potato (RAP)	Amount of water required for potato production per hectare per year	N/A	m^3/(ha*yr)	Parameter
Average water requirement for tamarillo (RAT)	Amount of water required for the production of tamarillo per hectare per year	N/A	m^3/(ha*yr)	Parameter
Average Water Requirement for Pasture (RAPAST)	Amount of water required for pasture production per hectare per year	N/A	m^3/(ha*yr)	Parameter
Utility coverage (CSP)	Percentage of coverage of public aqueduct services in the area	N/A	%	Parameter
Water requirements per person (RAHAB)	Amount of water required per person to lead a decent living condition	N/A	m^3/(da*hab)	Parameter
Water requirement per head of cattle (RAVACA)	Amount of water required in the basin for milk production	N/A	m^3/(da*vaca)	Parameter

VARIABLE	DEFINITION	EQUATIONS	UNITS	TIPE OF VARIABLE
Initial awareness value		N/A	habitantes	Parameter
Awareness delay		N/A	yr	Parameter
People impacted		N/A		Parameter
Efficiency of the environmental authority		N/A		Parameter
Institutional trust		N/A		Parameter
Articulation between policies		N/A		Parameter
Participation in decision-making		N/A		Parameter
Community participation		N/A		Parameter
Leadership		N/A		Parameter
Efficiency of private actors		N/A		Parameter
SRP initial		N/A		Parameter
Presence of livestock institutions		N/A		Parameter
Presence agricultural institutions		N/A		Parameter
Presence institutions conservations		N/A		Parameter
Agricultural motivation		N/A		Parameter
Livestock motivation		N/A		Parameter
Awareness increase		N/A		Parameter
Weight community participation (WPC)		N/A		Parameter
Weight leadership (WL)		N/A		Parameter
Weight Community organizations activity (WAOC)		N/A		Parameter
Weight efficiency of the environmental authority in policy compliance (WWEAA)		N/A		Parameter
Weight efficiency of private actors (WEPA)		N/A		Parameter

VARIABLE	DEFINITION	EQUATIONS	UNITS	TIPE OF VARIABLE
Weight acceptability (WAPC)		N/A		Parameter
Weight articulation between policies (WAEP)		N/A		Parameter
Weight articulation between actors (WAEA)		N/A		Parameter
Weight efficiency of the environmental authority in articulation between actors (WEAA in AEA)		N/A		Parameter
Weight Forest attractiveness (WAB)		N/A		Parameter
Weight institutional trust (WCI)		N/A		Parameter
Weight participation in decision-making (WPTD)		N/A		Parameter
Weight economic restrictions (WRE)		N/A		Parameter
Weight erosion (WE)		N/A		Parameter
Weight water (WW)		N/A		Parameter
Tradition associated with conservation (MA)	Tradition level of the area associated with conservation. This variable is related to the non-use values of the ecosystem services provided by the forest land cover.	$MA = Graphlinas\,(\frac{Sen(t)}{P_t}(1 + SRPn),\ [0,5])$	N/A	Auxiliar
Population – Zone 1 (HAB_1)	Number of inhabitants by area	$HAB\_1(t) = HAB\_1(0) + \int_0^t Change\ in\ population\ dt$	hab	Level
Population – Zone 4 (HAB_4)	Number of inhabitants by area	$HAB\_4(t) = HAB\_4(0) + \int_0^t Change\ in\ population\ dt$	hab	Level
Agriculture water demand	Amount of water required in the basin for the Agriculture sector	$(RAP * HP) + (RAT * HT) + (RAPAST * P)$	m^3/yr	Auxiliar

VARIABLE	DEFINITION	EQUATIONS	UNITS	TIPE OF VARIABLE
Water demand for domestic consumption	Amount of water required in the basin for the domestic sector	$HAB * CSP * RAHAB$	m^3/yr	Auxiliar
Livestock water demand	Amount of water required in the basin for the livestock sector	$RAVACA * IB$	m^3/yr	Auxiliar
Social Risk Perception (SRP)		$SRP\ (t) = SRP(0) + \int_0^t \ (C_{SRP})dt \quad (1)$	-	Level
Sensitized people (Sen)		$Sen\ (t)\ =\ Sen\ (0) + \int_0^t \ (TRS)\ dt \quad (2)$	hab	Level
Change in the SRP (C_{SRP})		$C_{SRP} = MAX\left[(A_{water} \times WW + A_{erosion} \times WE)\frac{Sen(t)}{P_t}\ ,\ -SRP(t)\right] \quad (3)$ A_{water}: Threat due to decrease in water, $A_{erosion}$: Erosion threat, P_t: Population, WW=0.65, WE=0,35	1/da	Auxiliar
Real awareness rate (RAR)		$RAR\ = MIN\left[\frac{AEA}{T_s}(EA), \frac{(P_t - Sen(t))}{1 \ll yr \gg}\right] \quad (4)$ EA: Environmental Awareness, Ts: Awareness time, P_t: Population	hab/yr	Auxiliar
Environmental motivation (MA)		$MA = Graphlinas\ (\frac{Sen(t)}{P_t}(1 + SRPn),\ [0,5])$ (5) SRPn: Social Risk Perception standardized, Graphlinas: is a Powersim specific language	-	Auxiliar
Articulation between actors (AEA)		$AEA = (AOC \times WAOC + EPA \times WEPA + EAA \times WEAA)\ CI \quad (6)$	-	Auxiliar

VARIABLE	DEFINITION	EQUATIONS	UNITS	TIPE OF VARIABLE
Command and control policy acceptances (APC)		AOC: Community organizations activity, WAOC: 0.25, EPA: Efficiency of private actors, WEPA: 0.3, EAA: Efficiency of the environmental authority, WEAA: 0.45, CI: Institutional trust<br>$APC = MAX\ [(AB \times WAB + PTD \times WPTD + CI \times WCI - RE \times WRE), 0]$ (7)	-	Auxiliar
Policy Compliance (CP)		AB: Forest attractiveness, WAB:0.35, PTD: Participation in decision-making, WPTD:0.15, RE: Economic Restrictions, WRE: 0.4, CI: Institutional trust, WCI: 0.1<br>$CP = APC \times WAPC + EAA \times WWEAA + AEA \times WAEA + AEP \times 0{,}20$ (8)<br>WAPC:0.3, AEP: Articulation between policies, WAEP:0.20, EAA: Efficiency of the environmental authority, WWEAA:0,35, WAEA:0.15	-	Auxiliar
Community organizations activity (AOC)		$AOC = MIN\left[(L \times WL + PC \times WPC) \times \frac{Sen(t)}{P_t}(1 + D\_SRPn),\ 1\right]$ (9)<br>L: Leadership, WL: 0.8, PC: Community participation, WPC: 0.2, D_SRPn: Delayed SRPn	-	Auxiliar

Summary of scenario and policy options simulation results in socio-cultural, economic and ES variables until 2040.

Table S2. Impacts of the combination of scenarios and policy options on some variables of SES. The percentages of variation are obtained from the comparison of the information between 2040 and 2015.

	**Combined 1**				**Combined 2**				**Combined 3**				**Combined 4**			
	**Zone 1**	**Zone 4**	**Zone 2**	**Zone 3**	**Zone 1**	**Zone 4**	**Zone 2**	**Zone 3**	**Zone 1**	**Zone 4**	**Zone 2**	**Zone 3**	**Zone 1**	**Zone 4**	**Zone 2**	**Zone 3**
**Water erosion**	-43,5%	-52,0%	6,6%	-1,9%	-41,1%	-50,7%	7,5%	9,3%	-44,4%	-53,2%	-11,2%	-13,1%	-41,9%	-51,9%	5,1%	7,7%
**Social Perception Risk**	59,6%	-54,5%	-1,0%	-1,0%	33,5%	-56,1%	-5,9%	-4,8%	88,1%	-50,1%	8,3%	8,6%	52,6%	-52,6%	-0,1%	-0,2%
**Intrinsic Motivation**	0,0%	-35,6%	0,0%	0,0%	0,0%	-55,0%	-10,0%	0,0%	0,0%	-33,1%	0,0%	0,0%	0,0%	-53,6%	-10,0%	0,0%
**Net Economic Benefits**	-138,4%	-129,3%	-117,4%	-116,9%	-140,0%	-130,3%	-120,7%	-121,0%	-154,7%	-141,6%	-136,7%	-135,7%	-157,0%	-143,1%	-143,7%	-144,4%

	**Zone 1 and 4**	**Zone 2 and 3**	**Zone 1 and 4**	**Zone 2 and 3**	**Zone 1 and 4**	**Zone 2 and 3**	**Zone 1 and 4**	**Zone 2 and 3**
**Concentration of SST (mg/l)**	-45,7%	1,6%	-43,6%	8,9%	-47,0%	-12,7%	-44,9%	6,9%
**Reduction Factor**	-56,9%	1,8%	-54,3%	9,9%	-58,6%	-14,2%	-56,0%	7,7%
